# Precision machine learning to understand micro-RNA regulation in neurodegenerative diseases

**DOI:** 10.3389/fnmol.2022.914830

**Published:** 2022-09-09

**Authors:** Lucile Mégret, Cloé Mendoza, Maialen Arrieta Lobo, Emmanuel Brouillet, Thi-Thanh-Yen Nguyen, Olivier Bouaziz, Antoine Chambaz, Christian Néri

**Affiliations:** ^1^Sorbonne Université, Centre National de la Recherche Scientifique UMR 8256, Paris, France; ^2^Université Paris Cité, MAP5 (Centre National de la Recherche Scientifique UMR 8145), Paris, France

**Keywords:** neurodegenerative disease, miRNA regulation, complex RNA-seq data, machine learning, precision analysis, shape analysis

## Abstract

Micro-RNAs (miRNAs) are short (∼21 nt) non-coding RNAs that regulate gene expression through the degradation or translational repression of mRNAs. Accumulating evidence points to a role of miRNA regulation in the pathogenesis of a wide range of neurodegenerative (ND) diseases such as, for example, Alzheimer’s disease, Parkinson’s disease, amyotrophic lateral sclerosis and Huntington disease (HD). Several systems level studies aimed to explore the role of miRNA regulation in NDs, but these studies remain challenging. Part of the problem may be related to the lack of sufficiently rich or homogeneous data, such as time series or cell-type-specific data obtained in model systems or human biosamples, to account for context dependency. Part of the problem may also be related to the methodological challenges associated with the accurate system-level modeling of miRNA and mRNA data. Here, we critically review the main families of machine learning methods used to analyze expression data, highlighting the added value of using shape-analysis concepts as a solution for precisely modeling highly dimensional miRNA and mRNA data such as the ones obtained in the study of the HD process, and elaborating on the potential of these concepts and methods for modeling complex omics data.

## Introduction

MicroRNAs (miRNAs) are short endogenously expressed non-coding RNA molecules that regulate gene expression by binding directly to the messenger RNA of protein coding genes. This layer of molecular regulation plays a pivotal role in several biological processes and is essential to brain development and homeostasis, regulating cell proliferation, differentiation, and apoptosis (Yang et al., 2013; Dahariya et al., 2019). Both the regulation of genes controlled by miRNAs and altered miRNA expression have been linked to several neurodegenerative diseases (NDs) such as Alzheimer’s disease and Parkinson’s disease. In the context of these two diseases, studies using blood samples and post-mortem brain tissue from patients have highlighted differential expression of miRNAs such as mir-1, mir-22p, mir-26b -3p, and mir-28-3p (Pritchard et al., 2012; Hu et al., 2016; Kumar et al., 2017; Garcia-Fonseca et al., 2021). Several studies also linked Huntington’s disease (HD) pathogenesis to miRNA regulation (Marti et al., 2010; Ghose et al., 2011; Jin et al., 2012). In particular, expression levels of the mir-200 family are altered in the cortex of mice with Huntington’s disease (HD) in the early stages of the disease. This deregulation affects a network of genes involved in neuronal plasticity and survival (Jin et al., 2012). In cellular models of HD, mir-146a, mir-125b, and mir-150 levels are decreased while mir-34b levels may be increased (Gaughwin et al., 2011). In amyotrophic lateral sclerosis, down-regulation of mir-9 (Haramati et al., 2010), up -regulation of mir-146a* and down-regulation of miRNAs 524-5p and 582-3p were reported in spinal cord compared to controls (Campos-Melo et al., 2013). These studies suggest that alterations of miRNA regulation could play a significant role in the responses to ND-associated genes (Quinlan et al., 2017), changing the molecular networks or signaling pathways involved in the control of cellular physiology and related phenotypes. It was also proposed that miRNA signatures found in biofluids such as cerebrospinal fluid and blood (Eacker et al., 2009; Sonntag, 2010; Ludwig et al., 2019) could provide a useful source of biomarkers for predictive diagnosis (Cortez et al., 2011; Noren Hooten et al., 2013; Machida et al., 2015).

However, despite a large amount of studies aimed at exploring the role of miRNA regulation in NDs, the identification of miRNAs that, on a systems level, might have a major influence on ND pathogenesis remains challenging. Since miRNA regulation is highly context dependent, part of this difficulty may relate to the lack of rich data, or sufficiently homogeneous data, that are required to get precise insights into the specificity of miRNA regulation, e.g., time series or cell type-specific data obtained in model systems time series data obtained from human biosamples. Part of the problem may also relate to the methodological challenges associated with accurately modeling miRNA-mRNA networks on a systems level.

To understand the role of miRNAs in disease pathogenesis, two main approaches may be developed. The first main approach relies on the analysis of miRNA data without considering mRNA data, an approach that was for example used to search for miRNA biomarkers. This analysis may be followed by listing the putative targets of the miRNAs retained in the model where information on targets is extracted from public databases (see section “Machine learning for modeling micro-RNA regulation in neurodegenerative research based on solely analyzing micro-RNA data”). Modeling miRNA regulation may indeed greatly benefit from databases in which information on putative miRNA-mRNA pairs have been generated using binding site data. However, several approaches have been proposed to predict miRNA targets based on binding sites. Some of the most commonly used criteria to predict miRNA targets include analysis of sequence complementarity between the “seed” region of a miRNA and the “seed match” region of a putative target mRNA, species conservation, thermodynamic stability and site accessibility (Menor et al., 2014). These methods can be classified into two categories. The first category comprises the so-called heuristic methods, namely methods having a tractable computational complexity but with no warranty of convergence toward a global optimum. The databases using this family of methods include TargetScan (Lewis et al., 2005) and mirSVR (Betel et al., 2010). The other category comprises machine-learning (ML) techniques such as decision trees, support vector machine and artificial neural networks. The databases mirMark (Menor et al., 2014), TarPmiR (Ding et al., 2016), TargetMiner (Bandyopadhyay and Mitra, 2009), TargetSpy (Sturm et al., 2010) and MiRANN (Rahman et al., 2012) are based on these algorithms. The second category makes use of ML methods. More sophisticated algorithms include deep learning methods such as for example DeepMirTar (Wen et al., 2018). Finally, to improve the coverage and robustness of the predictions of miRNA targets, some studies developed combinatorial ensemble approaches (Davis et al., 2017). For a review about the computational methods for miRNA targets prediction based on biological feature (see Min and Yoon, 2010). All these methods (Heuristics or ML) generate very large number of hypotheses, available in databases, providing putative targets for thousands of miRNAs. A common issue in all these methods that greatly limiting biological precision is that the number of possible targets for a single miRNA can be very large, requiring extensive validation studies.

**FIGURE 1:**
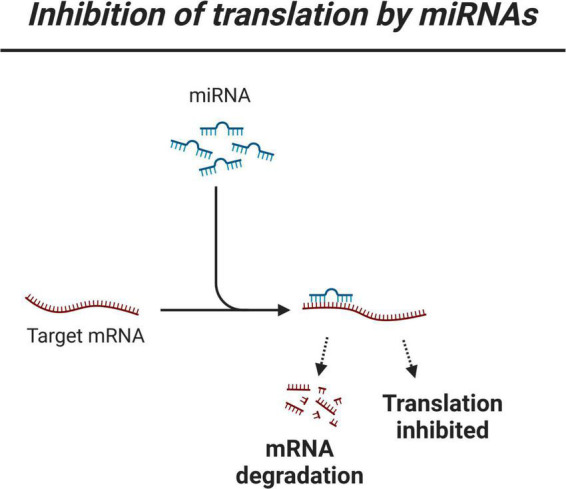
Simplified view of miRNA regulation of gene expression.

Another strategy for predicting miRNA targets is to analyze miRNA and mRNA expression levels to select negative correlations. The miRNAs may indeed regulate gene expression by blocking translation, though should the complementary sequences between miRNAs and their targets be good enough, the transcript may be degraded (Figure 1). The assumption is that these phenomena may translate into an inverse relationship between the expression level of a miRNA and its best matching mRNA target. Since miRNA regulation is a highly dynamic and context-dependent process, the value of using expression data is that these data may properly reflect molecular dynamics, which may then be complemented by using binding site data. Thus, a second approach is to infer miRNA regulation by using expression data that describe miRNA levels and mRNA levels. The conclusions obtained by using the first approach might remain significantly limited because miRNA databases usually contain a large number of targets for a given miRNA. The second approach may overcome this limitation, but this approach is usually more challenging to use (see below section “Machine learning for modeling micro-RNA regulation in neurodegenerative research based on analyzing micro-RNA and mRNA data”). Below, we review the main families of expression-based ML methods for the analysis of miRNA regulation in ND conditions, highlighting recent data that directly link miRNAs and NDs, also highlighting recent progress in better predicting the role of miRNA regulation in ND conditions thanks to the analysis of highly dimensional miRNA and mRNA data.

## Machine learning methods used to interrogate micro-RNA regulation in neurodegenerative diseases

ML methods are approaches from the fields of computer science and artificial intelligence (AI), designed to learn from the data. These technologies require large data sets, that enable the creation of statistical models. The advent of high throughput sequencing techniques has enabled the generation of such large datasets, thus enabling ML to gain momentum in the fields of medicine and biology, where it allows to analyze and integrate large data of RNA- and microRNA- sequencing. These methods can be divided into two main classes of algorithms: supervised learning algorithms and unsupervised learning algorithms. The former class involves feeding the algorithm with labeled data that, after training, enable unknown information to be labeled based on the patterns learned from the data used for training, namely the training set, whereas the purpose of the latter class is to classify the data, based on similar patterns in the data itself (Carpenter and Huang, 2018; Garcia-Fonseca et al., 2021).

In this section, we briefly present some of the mostly used families of algorithms for the analysis of miRNA expression data in the context of ND research (Table 1).

**TABLE 1:** Machine learning methods used for research on the association between miRNAs and neurodegenerative diseases.

Methods	Positive aspects	Negative aspects	Examples of use in the context of miRNA analysis
**Ridge**	Reduces the impact of variables that are not important for the prediction	Doesn’t eliminate irrelevant variables	PMID: **26947266**
**Lasso**	Reduces overfitting by adding a penalty to coefficients the model overemphasizes and eliminates them	Doesn’t take into account multicollinearity in the model and could eliminate relevant independent variables	PMID: **34048985**
			PMID: **33316739**
			PMID: **21743061**
**Elastic net**	Combines both Lasso and Ridge aspects: It eliminates some variables while reducing the impact of some other variables	Computationally more expensive than LASSO or Ridge	PMID: **35113902**
			PMID: **29513198**
**Decision tree**	Very simple to understand and visualize	Subject to overfitting	PMID: **26649272**
		Doesn’t work well with imbalanced data	
		Very different trees can be generated if a small chance in the data is made	
**Random forest (RF)**	Can deal with imbalanced datasets and missing data	The number of nodes in decision trees will grow exponentially with depth	PMID: **29056906**
	Being an ensemble of decision trees, overfitting is not a problem	The prediction needs to be uncorrelated	PMID: **23922946**
**Gradient boosted decision trees (GBDT)**	More accurate than RF	Sensibility to outliers	PMID: **32604706**
	Doesn’t need bootstrap sampling like RF	Overfitting can be a problem when too many trees are added	PMID: **35051896**
**Support vector machine (SVM)**	Works well with 2D, 3D, or higher dimensions	Computationally more expensive for larger datasets	PMID: **29275361**
			PMID: **24417022**
	Outliers have less impact on the prediction since the hyperplane is influenced by the support vectors (data points closer to the hyperplane)	Works poorly if the dataset has overlapped classes	PMID: **34442108**
**Artificial neural networks (ANN)**	Work very well with huge amount of data	Can be quickly computationally and time consuming	PMID: **30504368**
	Can handle unstructured data	Big dependence on the training data, so overfitting can be a problem	PMID: **22349176**
			PMID: **30519653**
**k-means**	Very simple algorithm to implement	Lack of robustness with big data analysis	PMID: **34879829**
		Choosing K can be difficult	PMID: **32493067**
		Doesn’t work well with imbalanced data or outliers	PMID: **22255820**
**Weighted correlation network analysis (WGCNA)**	Retains connectivity of nodes	Can lack biological precision	PMID: **32699331**
			PMID: **34225819**
**Bayesian network**	Can handle missing data and avoid overfitting	Need for sensitivity analysis, to be applied to the outcome	PMID: **23690582**
			PMID: **32368197**

### Supervised learning

#### Regression

The aim of this type of supervised learning is to predict the output value from a set of input values. The algorithm is first trained, to learn how to predict the output value, then the model can be applied to predict the output value of new data. When there is enough data, a good practice is to train the algorithm to predict the output on a subset of data (training dataset) in order to be able to verify that the algorithm is indeed capable of predicting the output of a subset of data (test dataset) whose output are known by the user but has not been provided to the algorithm. An alternative approach when the dataset is small is to use leave-one-out cross validation. The main purpose is to determine a continuous variable based on the data, but it can also be used for features selection. When there is a large amount of predictors, it might be useful to use stepwise subset selection methods in linear regression, that is to progressively add (forward selection) or remove (backward elimination) predictor variables and to select the model that achieves the best performance (Zhang, 2016). In the context of big data, penalized regressions, Ridge or Lasso, might be used. Ridge regression shrinks the regression coefficients, to put the coefficients of the less important variables close to zeros. The shrinkage of the coefficients is achieved by penalizing the L2 norm (square root of sum of the square) of the vector of coefficients (Hoerl and Kennard, 1970; le Cessie and van Houwelingen, 1992). Lasso stands for Least Absolute Shrinkage and Selection Operator. This methods penalizes the L1-norm (sum of the absolute values), it has the effect of effect of forcing some of the coefficients, those with a minor contribution to the model, to be exactly equal to zero (Tibshirani, 1996). Thanks to this feature, Lasso may also be used as a variable selection method that might achieve best performances compared to subset selection methods (Morozova et al., 2015). Finally, Elastic Net produces a regression model that is penalized with both the L1-norm and L2-norm (Zou and Hastie, 2005). Therefore, ElasticNet effectively reduces coefficients (like in ridge regression) and puts some of them exactly to zero (as is Lasso) (Leach et al., 2022).

#### Classification

Classification aims to identify some patterns from a set of data that allows to predict their label. The goal is to make the model able to automatically label a new sample using the patterns established from the training set. In the context of association of dysregulated miRNAs with NDs, this type of method may be used, for example, to classify a sample as healthy or sick based on miRNA expression level. Some such algorithms are Decision Trees (Breiman, 1984). Decision jungle (Shotton et al., 2013), Random Forests (Breiman, 2001), Support Vector Machine (Cortes and Vapnik, 1995), and k-NN classification (Altman, 1992) among others. A decision tree is a directed graph in which each node corresponds to a “test” on an attribute, each branch represents the result of the test, and each final node provides predicted the label. The paths from root to leaf are classification rules. Random forest (RF) and gradient boosted decision trees (GBDT) (Zhang and Jung, 2021) are two widely used machine learning algorithms, based on decision trees. In both methods, a weak learner (decision tree) combination is used to obtain a more robust (but less interpretable) model. The major difference lies in the fact that RF is built using the so-called bagging method in which each decision tree, grown on a subsample of the entire data-set, is used as a parallel estimator (Breiman, 1996). For a classification task, the final result is obtained by vote on all decision trees. In the case of a regression task, the final result is obtained by computing the mean value of all predictions. On the other hand, GBDT make use of boosting techniques in order to create an ensemble learner (Friedman, 2001). In this case, decision trees are connected sequentially (i.e., in series) to build the final model. Thus, while bootstrapping is of major importance to the success of a RF model, which highly depends on using uncorrelated decision trees, in GBDT, each decision tree is fitted on the residuals from the previous tree, which prevents the trees to be correlated. As a second consequence, GBDT is subject to overfitting if too many trees are used, while adding too many trees to a forest just increases the computational cost of the model, without increasing the risk of overfitting. A major issue of RF is that, given enough data are available to the analysis, the number of nodes in decision trees will grow exponentially with depth. Decision jungles have been developed to overcome this problem (Shotton et al., 2013). Support Vector Machine (SVM) perform classification task by finding the best hyperplane, that is, the one that best separates the data. SVM are based on two key ideas: the notion of maximum margin and the notion of kernel function. The margin is the distance between the separation boundary and the nearest samples. In SVM, the separation boundary is chosen as the one that maximizes the margin. To deal with data that are not linearly separable, the second key idea of SVM is to transform the representation space of the input data into a higher dimension space in which it is likely that linear separation exists. This is achieved by a kernel function. New examples are then mapped into that same space and predicted to belong to a category based on the side of the separation boundary in which they fall in Chang and Lin (2011).

Finally, Artificial neural networks (ANN) are another class of non-linear classifiers. These classifiers are named ANN as these models mimic the way that a signal is transmitted and processed by biological neural networks (Abiodun et al., 2018). The ANN are able to deal with complex interactions between observed variables in order to predict an outcome. The basic unit of an ANN is called an artificial neuron. These neurons are represented by connected nodes. Each node can transmit a signal to other neurons. An artificial neuron receives a signal, processes it and, depending on the results, sends a signal to the neurons connected to it. The input signals transmitted to a neuron at every incoming connection is a number. The output of the neuron is computed by a non-linear function apply to the sum of its inputs. The strength at connections are determined by weights that adjusts during the learning process. In order to manage the complex relationships between variables, neurons can be grouped into layers. Different layers can perform different transformations on their inputs. Signals travel from the input layer to the final layer, giving the desired output (labels if a classification task is performed). Depending on the type of neural network considered, the signal can cross the same layer several times.

### Unsupervised learning

#### Clustering

With regard to unsupervised learning, there is no known desired output. Thus, this family of algorithms tries to find clusters or groups in the unlabeled data based on their similarity. The elements grouped in the same cluster are expected to share similar features and be closer together than to elements from other clusters. For instance, k-means algorithms group the input data into a user defined number of clusters (k). The first step of the algorithm consists of randomly choosing as many points in the variable space as there are clusters to be identified. These points are called centroids and are used to define the clusters. The data are grouped according to their proximity to the different centroids, that adapt to cluster the data such that the data point in a given cluster share more similarity than with points from other clusters. A limitation of these methods is the dependence on initial centroids, chosen at random, which can make the algorithms converge toward a different partition of the data space, particularly if the number of data to be grouped is high or if the data are noised.

#### Network inference

The purpose of these methods is to perform inferences and predictions about a biological network. A network is a set of nodes and a set of directed or undirected edges connecting the nodes. It exists a wide range of type of biological network, where edges can be protein-protein interactions or genetic interactions. Complete true biological networks are rarely known, but they can be statistically inferred from a similar behavior of nodes in terms of gene expression, protein levels or metabolite levels across conditions (Jansen et al., 2003; Jia et al., 2017; Zheng et al., 2021). Several methods can be used to generate biological networks, notably using gene expression data. Among the methods that have so far been used, to analyze the role of miRNAs in NDs, correlation networks are becoming increasingly popular in order to analyses the role of miRNAs in NDs, correlation networks becoming increasingly more popular (Langfelder et al., 2018; Kakati et al., 2019; Megret et al., 2020; Zhang et al., 2021). For instance, weighted gene co-expression network analysis is a systems biology method for describing the correlation patterns among genes across microarray samples. Weighted correlation network analysis (WGCNA) can be used for finding clusters (modules) of highly correlated genes. Expression profiles of genes in each cluster are summarized using a representative profile called the eigengene (Horvath and Dong, 2008). This method allows to overcome the lack of robustness of clustering methods, thanks to the use of consensus modules, but it generates large modules, which may impair the strength of gene prioritization, even when considering hub genes (i.e., highly connected genes) (Botia et al., 2017). Finally, Bayesian approaches are network methods that provide probabilistic models with a causal relationship between entities. A Bayesian network is a probabilistic graphical model that represents the conditional dependencies between the variables via a directed acyclic graph (DAG). This approach allows to introduce prior knowledge in the model via the definition of the graph and the prior chosen for the conditional relationships. This particularity enables prior knowledge and the information contained in the data to be considered altogether. However, the need for rules to build the model may increase with the number of features. When dealing with big data sets, there is a need for robust Bayesian analysis, that is Bayesian analysis with some level of sensitivity analysis, to be applied to the outcome of Bayesian inference (Berger et al., 1994). In other words, a model is robust if it does not depend too much on the assumptions and calculation inputs on which it is based.

## Machine learning for modeling micro-RNA regulation in neurodegenerative research based on solely analyzing micro-RNA data

Dementia with Lewy bodies (DLB) is the second most common sub-type of neurodegenerative dementia following AD. Shigemizu et al. (2019) compared four Supervised ML classifiers for their performance in predicting the DLB status of data collection of Japanese individuals (*n* = 478), based on miRNA expression data of serum samples, including penalized regression, SVM, RF and GBDT. Interestingly, GBDT was found to achieve the highest AUC in this study. GBDT retained 7 miRNAs mir-3122, mir-6861, mir-4298,mir-6088, mir-4728, mir-5698, and mir-1909) with the highest feature importance. The authors then looked at the 423 genes that were predicted as targets of these miRNA in miRDB (Chen and Wang, 2020). Six signaling pathways were found to be enriched in these 423 genes including: protein kinase A signaling (21 genes), ERK/MAPK signaling (14 genes), molecular mechanisms of cancer (20 genes), p38 MAPK signaling (10 genes), glucocorticoid receptor signaling (18 genes), and docosahexaenoic acid (DHA) signaling (6 genes), with a *q*-value < 0.05. Although these pathways may be viewed as relevant to DLB, miRNA data were collected from serum samples. Additionally, this study illustrates how the sole use of miRNA databases as a way to connect miRNAs with targets may lead to poor biological precision, particularly when one miRNA is connected with a very large number of potential targets.

Hearing loss is the most common ND worldwide. To highlight the miRNAs that can be used as biomarker to predict sensorineural hearing loss (SNHL) and severity of sensorineural hearing loss, Shew et al. (2019) competed four supervised ML classifiers. The miRNAs expression data were collected from perilymph in 16 patients, a multi-class decision forests, a decision jungle, a logistic regression and neural networks was trained, to predict SNHL and severity of (SNHL) with miRNA expression data. A leave-one-out cross validation approach was used to test the model obtained. The misclassification error for each model was: decision forest, 0%; logistic regression, 8.33%; decision jungle, 25%; and neural network, 41.67%.

The permutation feature of importance has been applied to the ML models in order to exhibit the most predictive miRNA and, then explore their functional role. In order to be considered as significant, a miRNA has to be used in the construction of, at least, two models. The most heavily weighted miRNAs used in the models to predict the severity of SNHL included mir-184, mir-660, mir-let-7a-5p, mir-3142, and mir-335. A search for anticorrelated genes in the inner ear with the expression level of the selected miRNAs was then performed, as well as a search for potential connections between these miRNAs and SNHL using Ingenuity Pathway Analysis (IPA) software. The key miRNA and their putative targets included mir-184, mir-660, and mir-let-7a-5p. There were no known interactions predicted using IPA software for mir-3142 and mir-335. In this study, the decision forest method achieved the best performance while decision jungle and neural network methods are poor predictors. In addition to the data set being very small, models achieving very poor performance were used in the selection of most significant miRNAs. Small dataset and poorly efficient predictors cast a doubt on reliability, and the rather large set of potential targets (e.g., 34 putative targets for mir-184) does not favor biological precision. This study illustrates again that the sole use of miRNA databases as a way to connect miRNAs with targets to make a hypothesis about the role of miRNA regulation in pathogenesis is highly speculative by design.

In another study by Gullett et al. (2020), four ML methods were trained to predict Montreal Cognitive assessment (MoCA) in healthy older adults solely based on miRNA expression data. The miRNAs were collected from blood in 115 typically aging older adults. The algorithms used in this study included boosted decision tree (BDT), RF, ML-based linear regression and stepwise selection based on AUC, and their performance were compared for predicting MoCA scores with miRNA expression data. The ability of these four models to predict other cognitive function measurements, such as NIHTB overall cognition, fluid cognition, and crystalized cognition, was also tested. In the prediction of MoCA score, the best performance in this study was achieved by BDT model, using only miRNAs. However, when using the other cognition rating scales, miRNA and other explanatory variables (clinical data, biological data, social factors) provided the best RF prediction of cognitive performance compared to either group of data alone. The two top miRNAs to predict cognitive performance in terms of average rank were mir-335-5p and mir-2110. Some other miRNAs were highly ranked predictors across two cognitive measures, including mir-181c-3p, mir-497-5p, mir-425-3p, and mir-221-3p.

These studies raised the possibility that decision tree forest, and particularly BDT, may be the best methods to highlight miRNAs as potential biomarkers in NDs. However, the source data used in these studies are restricted to miRNA data, with no data produced for analysis of mRNAs, which does not allow robust conclusions to be raised on the role of the retained miRNAs in disease pathogenesis.

## Machine learning for modeling micro-RNA regulation in neurodegenerative research based on analyzing micro-RNA and mRNA data

Some approaches make use of gene expression profiles to select putative targets among those retained targets based on binding sites. Such approaches include the use of Bayesian analysis such as GeneMiR++ (Generative model for miRNA regulation), that accounts for patterns of gene expression using miRNA expression data and a set of candidate miRNA targets. This method has been applied to obtain several sets of causal networks, built upon different subsets of the transcriptomics profiling, proteomics profiling and behavioral profiling data in the brain of the allelic series of Huntington disease knock-in mice (Hdh mice). Yet, the optimal fit between miRNAs and putative targets retained by Bayesian causal inference may be biased because the network of causal interactions is large and heterogeneous, involving miRNA-to-miRNA, mRNA-to-mRNA, and mRNA-to-miRNA interactions in addition to direct miRNA-mRNA interactions (Le et al., 2013). To only retain the miRNA-to-putative targets interactions, Bayesian networks may be filtered using information from external databases on miRNA binding sites (Zhang et al., 2014). However, filtering the network can exacerbate the problem of miRNA effect sizes by aggregating all branches from one miRNA. Weighted gene correlation network analysis (WGCNA) is another expression-based network inference approach. For example, WGCNA was used to search for negative correlations between miRNA co-expression modules and target co-expression modules in the study of miRNA regulation in hepatitis C (Peng et al., 2009) and in the brain (striatum and cortex) of Hdh mice (Langfelder et al., 2018). The first step in this study was to search for the most deregulated miRNAs, which retained 480 dysregulated miRNAs in striatum, cortex, cerebellum and liver. The second step was to evaluate possible connections between microRNA and mRNA expression levels, which was performed by using previously published WGCNA mRNA modules (Langfelder et al., 2016). The enrichment of these mRNA modules in putative [as inferred using (Betel et al., 2008), microCosm (Griffiths-Jones et al., 2008), targetScan (Lewis et al., 2005)] and validated [as inferred using mirTarBase (Hsu et al., 2014)] targets of deregulated miRNAs was then evaluated. This latter step retained 124 striatal and 10 cortical miRNA-mRNA modules of interest with enrichment *p*-values less than 0.05. The third step was to retain the links where the miRNA expression levels are negatively correlated with the mRNA module eigengene. About half of these links were found to exhibit such negative correlations and the mRNAs retained in this category were subjected to biological content analysis to identify the biological processes and pathways that may be impacted. The miRNAs of strong interest that were eventually retained in this study are shown in Table 2. The use of ML made in this study roughly paired miRNAs with mRNA modules that are large (up to 838 mRNAs). This approach is interesting in terms of modeling, but the information about CAG repeat- and age-dependent changes that is contained in the data was not fully exploited to define in a very accurate manner the potential match between miRNAs and targets and to reduce data complexity accordingly.

**TABLE 2:** Comparison of miRNAs retained in the striatum of HD model knock-in mice using a WGCNA-centric approach (Langfelder et al., 2018) or the MIRAMINT pipeline (Megret et al., 2020).

Mir1247	Miramint
Mir132	Miramint, WGCNA
Mir133b	Miramint
Mir139	Miramint, WGCNA
Mir187	Miramint
Mir1b	Miramint
Mir20b	Miramint
Mir222	Miramint, WGCNA
Mir299b	Miramint
Mir3102	Miramint
Mir363	Miramint
Mir378b	Miramint
Mir484	Miramint
Mir673	Miramint
Mir128-1	WGCNA
Mir212	WGCNA
Mir218	WGCNA
Mir181d	WGCNA
Mir128-2	WGCNA
Mir221	WGCNA
Mir29a	WGCNA
*Mir181a-1*	WGCNA
*Mir186*	WGCNA
*Mir320*	WGCNA
*Mir340*	WGCNA
*Mir543*	WGCNA
*Mir186*	WGCNA
*Mir363*	WGCNA

Network inference approaches such as Bayesian and WGCNA approaches provide useful insights on the characteristics of miRNA regulation, however, they can be prone to aggregating a large number of assumptions around strongly deregulated entities. Ultimately, this problem may lead to a lack of biological precision, limiting the level of precision that is needed to enhance data prioritization and minimize biological follow-up studies.

To address this problem, we applied a workflow in which a network-based analysis for reducing data complexity precedes a RF analysis for selecting explanatory variables (i.e., miRNAs best explaining targets, with a *P*-value computed for each predictor variable). Importantly, we used as a final step a surface-matching analysis for a more comprehensive level (shape-based) of negative correlations between the expression patterns of miRNAs and their putative targets. The introduction of the “surface-matching” procedure is based on the idea that we can consider data as shapes (e.g., expression curves, expression surfaces) and these shapes can be tested for similarity across conditions to delineate clusters at high precision.

**FIGURE 2:**
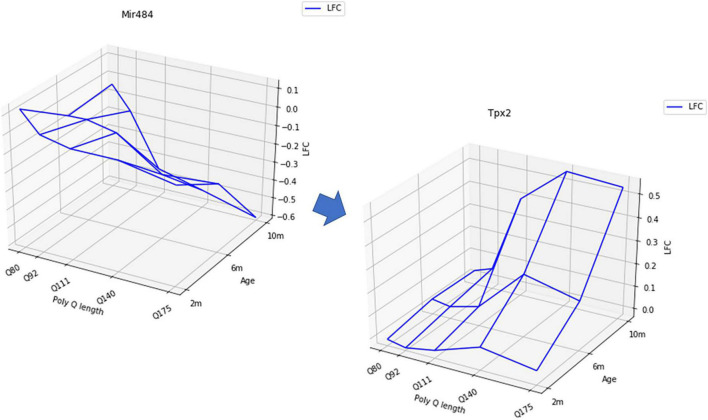
Examples of a mRNA expression surface negatively correlated with a miRNA expression surface in the striatum of HD mice.

The pipeline integrating WGCNA, RF and surface-matching (see Figure 2) (called MIRAMINT) was applied to data collected in the striatum and cortex of HD model knock-in mice across 6 CAG repeats lengths and 3 age points (Megret et al., 2020). This approach led to the conclusion that, on a global level, miRNA regulation may have a limited role in the HD process, contrasting with previous analyses of the same datasets (Langfelder et al., 2018). This approach also led to selecting a small number of robust miRNA-mRNAs that in the striatum could be associated to the disease process, notably implicating Jak-STAT signaling, Th1 and Th2 cell differentiation, ether lipid metabolism and N-glycan biosynthesis signaling pathway (Table 2). However, shape analysis concepts in MIRAMINT were used in a simple manner and as a refinement step after data reduction and feature selection. Other methods in which shape analysis is at the center of the analysis of complex omics data may be used to interrogate miRNA regulation. Noticeably, *Geomic* analysis, a shape deformation analysis that was developed to understand the dynamics of compensatory and pathogenic responses to mutant huntingtin in specific striatal cell types of the HD model knock-in mice (Megret et al., 2021) could be used for modeling miRNA and mRNA data. Shape analysis methods such as optimal transport may also be used for analyzing miRNA and mRNA data, as currently illustrated by the analysis of the best mirroring relationships between miRNA and mRNA expression levels in the striatum of HD model knock-in mice (Nguyen et al., 2021). In this work, optimal transport is first used to derive a similarity matrix that quantifies the proximity between each mRNA to each miRNA. In a second step, high values of similarity are selected while controlling the maximum number of possible matches between mRNAs and miRNAs. This weighted transformation optimal transport (WTOT)-matching algorithm yielded a total of 7,519 matched sets (mRNA, miRNA) between 4234 mRNAs and 1043 miRNAs. In this approach, shape analysis was further refined by specifically aiming to retain monotonous mRNAs profiles using a first criterion and peaked mRNAs profiles using a second criterion. The first criterion yielded 212 mRNAs matched with 122 miRNAs, and the second criterion yielded 43 mRNAs matched with 68 miRNAs. The relatively small number of mRNAs that were matched at high precision to the miRNAs profiles in each shape class suggests that WTOT-based methods are promising shape-analysis methods for reducing the complexity of omics data. Indeed, obtained a limited number of matched pairs allow for an easy data prioritization that is of high importance to be able to test experimentally the best hypotheses. In addition, large sets of matched pairs including a large number of mRNAs, risk involving a large number of biological signaling pathways, make it difficult to reach a claim about the major biological mechanisms under miRNAs regulation. Since methods are needed to perform data prioritization, having a ML pipeline that includes a data prioritization step might be helpful.

From a methodological standpoint, searching for perfect matching might be not the most relevant assumption. Indeed, the shape-analysis procedure was made possible using Log2-fold-change (LFC) values in different conditions. For each gene, instead of a list of count data in different conditions, the data take the form of a unique value (LFC: z-axis) in each condition (in this data set, three ages: *x*-axis and four polyQ length: *y*-axis) (Figure 2). It seems reasonable to consider the LFC instead of individual expression levels to have a global view of the deregulation of mRNA that might be due to miRNAs deregulation. But, since the calculation of the LFC averages the expression on all the samples in the same condition, it is not obvious that, when a mRNA is under the regulation of a miRNA, their LFC surfaces must be perfectly anti-correlated. This raises the question of the choice of the threshold to consider that two surfaces are “sufficiently” negatively correlated and that this correlation may reflect biological regulation. This threshold is probably below that required in statistical terms to conclude to a significant anti-correlation. This leads to very stringent criteria, which do not necessarily select the “best” hypotheses, since there is no reason to think that the surfaces must be perfectly anti-correlated. In addition, whatever the method considered, a major issue of the use of expression data to search for negative correlations between miRNA and mRNA reduces the relevance of the analysis to the case where the mRNA transcript is degraded by the miRNA, missing all the regulation due to the inhibition of transcription (Figure 1). It is worth to notice that the use of LFC instead of individual expression levels allow to filter the genes (miRNA or mRNA) that are significantly dysregulated.

## Concluding remarks and future perspectives

On a global level, except to some miRNAs that are often recruited across studies of NDs using ML on large miRNA expression data sets, such as miRNA 132 (Langfelder et al., 2018; Megret et al., 2020), miRNA 221 (Langfelder et al., 2018; Gullett et al., 2020) or miRNA 335 (Shew et al., 2019; Gullett et al., 2020), few overlaps have been observed between studies. Since miRNA regulation is highly context-dependent, it is not surprising that miRNA patterns and signatures may significantly vary from one disease to another. However, it is interesting to note that when the role of miRNAs in a given data set is studied by using different approaches, conclusions may also be significantly different, with little overlap between miRNA-target pairs retained across approaches (Langfelder et al., 2018; Megret et al., 2020). This phenomenon is common to all analyses of big data using different ML methods. A major difficulty is that the use of unsupervised methods makes it difficult to evaluate the accuracy of the conclusions. In this context, current studies suggest that the added value of shape matching for the analysis of complex omics data, particularly when omics data are sufficiently dimensional, is to greatly reduce the number of resulting hypotheses while tipping the balance toward biological relevance as observed with the analysis of miRNA regulation in mouse models of HD pathogenesis. However, several metrics can be used to compare the shapes of highly dimensional omics data, including shape-deformation- and optimal-transport-based metrics, and several methods can also be used to cluster data. The statistical tests to be used for selecting significant matching depend of the retained metrics. In the case of a simple correlation, the *p*-value might be a relevant criterion. In the case of a more refined metric, such as shape deformation or optimal transport, the user has to choose a threshold reflecting that the distances between a miRNA and its putative target(s) are “small.” The distribution of the distances over all pairs might also be used. Permutation analysis to test for matched pairs that are truly specific to the data might be an option, but, in practice, the computational cost associated with performing permutation analyzes might be a limitation. In all cases, the choice of a shape-based metric allows a great level of refinement in the analysis of the data, providing a high degree of precision in matching miRNA and mRNA expression profiles. Noticeably, the requirement for perfectly negative correlations might be not the best criteria for retaining miRNA-mRNA pairs of interest, and this by the way may apply to searching for positive correlations, for example between gene or protein expression profiles. Current data suggest that properly designed shape analysis is a powerful approach to enhance the precision of data modeling and to improve biological accuracy in studies of complex omics data (Megret et al., 2020, 2021; Nguyen et al., 2021). Another interesting feature of shape analysis is that it has a wide range of applications for the analysis and integration of omics data and that specific (evidence-based) assumptions on the biological behavior of the variables of interest can be used for building shape models. As such, shape analysis is emerging as a promising approach for data analysis in biology and disease. However, a challenge common to all shape-based methods is to identify the right setting between perfect and unperfect shape-matching, and this challenge applies to omics data and the construction of shape models that may best account for the phenomenon that is interrogated (e.g., molecular regulation, cell biology, or disease progression). Considering that mRNA biosynthesis and degradation is not fully dependent on miRNA regulation, and considering that several biological processes may be regulated by multiple independent factors, with stochasticity involved, perfect shape-matching may be not the best criterion for retaining molecular players into a model as this could lead to a loss of relevant information. On the other hand, retaining shape-matching events that are too imperfect might favor a decrease in the discriminative power and biological precision of the resulting model. With regard to miRNA regulation, it would be interesting to have reference data, that is a large number of biologically validated miRNA-mRNA pairs, in specific cellular contexts, in order to have a reliable benchmark criterion for testing the effectiveness of predictive methods, which highlights the value of quantitative biology data in research on miRNA regulation. Future studies involving a larger number and more diverse array of datasets will provide important insights into the optimization of shape analysis methods for precision machine learning and for making sense of complex omics data.

## Author contributions

LM and CN wrote the manuscript. CM helped with literature searches and designed the tables. MA, EB, T-T-YN, OB, and AC critically reviewed the manuscript on either a biological and clinical (EB) and machine learning level. All authors contributed to the article and approved the submitted version.
